# Non-Targeted Metabolomics Reveals the Metabolic Differentiation of Rice from Adjacent Small-Scale Producing Areas and Its Response to Climatic and Soil Factors

**DOI:** 10.3390/foods15142499

**Published:** 2026-07-15

**Authors:** Xianxin Wu, Zeting Li, Tianshu Peng, Lina Li, Qiujun Lin, Guang Li, Chunjing Guo, Qingchuan Liu, Jianzhong Wang

**Affiliations:** 1 Institute of Agricultural Quality Standards and Testing Technology, Liaoning Academy of Agricultural Sciences, Shenyang 110161, China; wuxianxin1225@163.com (X.W.); tspenglaas@163.com (T.P.); lln231118@163.com (L.L.); linqiujun85@163.com (Q.L.); lngtgh@163.com (G.L.); guocj464@163.com (C.G.); 2 Fushun Inspection & Examination Certification Center, Fushun 113000, China; lizt2026@163.com; 3 Fushun County Agricultural and Rural Development Service Center, Fushun 113009, China; 15941310656@163.com

**Keywords:** rice, characteristic metabolic markers, climate, soil chemical environment, geographical traceability

## Abstract

Geographical traceability of rice is critical for authenticity identification and quality control, yet it poses considerable challenges for tracing origins in adjacent small-scale producing areas. To explore the causes of metabolic differences and geographical traceability potential of rice from adjacent small-scale producing areas, non-targeted metabolomics combined with multivariate statistical analysis was employed to systematically investigate the metabolic profiles of rice from Panjin (PJ), Donggang (DG) and Yingkou (YK) in Liaoning Province. The characteristic metabolic markers for each producing area were screened, and the effects of climatic and soil factors as well as their interactive effects on grain metabolite composition were elucidated. The results showed that the partial least squares-discriminant analysis (PLS-DA) model established based on differential metabolites achieved acceptable discrimination among rice samples from the three regions. With variable importance in projection (VIP) > 2.0 as the screening threshold, the core characteristic markers of each producing area were determined: PJ is LPC 17:2, LPA 18:3, D-(+)-Arabitol, DG is 2′-Deoxyadenosine, LDGTS 18:2, and YK is LPE 17:2; the markers are mainly primary metabolites, including lipids, sugar alcohols and nucleotides. Sunshine duration, air humidity, wind conditions and soil layer temperature were highly correlated climatic drivers responsible for metabolic differentiation, and characteristic metabolic markers from different producing areas exhibited distinct meteorological response patterns. Soil physicochemical properties and mineral elements significantly affected the differential accumulation of metabolites, among which soil Sr element and organic matter exhibited crucial indicative significance for metabolic variation of rice in adjacent regions. Multi-factor interaction analysis verified significant synergistic coupling effects between regional climate and soil environment. Meteorological factors, including sunshine, wind and soil temperature, together with soil chemical factors involving organic matter, pH, Sr, K and Ca, were identified as core driving factors for the spatial differentiation of region-specific rice metabolites. The present study provides theoretical support at the metabolic level for the construction of a small-scale rice geographical traceability system and the mechanism research on the regional quality formation of rice.

## 1. Introduction

Rice, serving as the staple food for nearly half of the global population, is a core carrier for energy supply, and its metabolite composition directly determines nutritional quality, flavor characteristics, and health value. In recent years, with breakthroughs in metabolomics technology, thousands of small-molecule metabolites in rice, including amino acids, carbohydrates, lipids, and secondary metabolites (e.g., phenolics, terpenoids, alkaloids), have been gradually uncovered [1,2], providing new insights into understanding rice growth and development, stress resistance mechanisms, quality formation, and environmental interactions. Against this backdrop, systematically exploring the regulatory mechanisms driving rice metabolite composition and identifying key environmental control factors are crucial for developing improved rice varieties that balance yield and quality. This is not only a critical scientific issue for enhancing the nutritional functionality of rice and meeting the demands of consumption upgrading but also a strategic imperative for addressing global climate change challenges and ensuring food security [3].

Rice metabolites represent the final products of the plant’s response to environmental stimuli during specific growth stages, and alterations in any single environmental factor can significantly modify their accumulation levels [4]. As a key factor affecting plant metabolite composition, water has been reported to significantly influence metabolic pathways in rice, including those of hormones, vitamins, amino acids and their derivatives [5]. After the function of phytochrome B, which mediates light absorption, was inhibited, the contents of free fatty acids and lysophosphatidylcholines in rice grains were significantly increased, while the levels of sugars, alcohols, amino acids and their derivatives, organic acids, phenolic acids, alkaloids, and nucleotides were decreased, confirming the crucial role of light in shaping the metabolite profile of rice [6]. The secondary metabolic synthesis pathways, amino acid biosynthesis, and glutathione pathway in rice have been reported to be closely related to temperature [7]. The levels of some elements in the soil are also important factors affecting the metabolic composition of rice. For example, when rice is subjected to cadmium stress, metabolic pathways such as carbohydrate metabolism, lipid metabolism, flavonoid biosynthesis, amino acid biosynthesis and metabolism, amino sugar and nucleotide sugar metabolism, and diterpenoid biosynthesis all undergo significant changes [8]. Another study has shown that mercury treatment significantly affected lipid metabolism and amino acid metabolic pathways in rice, and it was found that the key metabolites responding to mercury accumulation were silymarin and 12-hydroxy lauric acid [9]. Furthermore, soil type and composition, metal elements, geographical origin, and other factors have also been identified as significant influences on plant metabolite synthesis and accumulation [10]. Given the sensitivity of metabolites to growth environments, the combined application of metabolomics technology and chemometric analysis has been widely adopted for geographical origin traceability of agricultural products [11]. Among various chemometric methods, supervised partial least squares analysis (PLS-DA) exhibits favorable performance in identifying rice geographical origins based on metabolomics data. For instance, the integration of metabolomics and PLS-DA has been successfully applied not only to distinguish brown rice from large-scale geographical regions, including China, Vietnam and India [12], but also to effectively trace the origins of rice produced in different provinces in China, such as Jiangsu and Heilongjiang [13].

However, most existing studies have focused on the isolated effects of single factors, while the interactions between climate and soil chemical environments and the mechanisms underlying the cascade regulation of metabolites remain unclear. Moreover, whether rice metabolic response patterns exhibit regional specificity across cultivation environments with small-scale geographical distances within the same province lacks systematic comparative analysis. Furthermore, genetic factors such as varietal differences, which influence the metabolic composition of agricultural products, confound the precise analysis of how environmental factors regulate rice quality formation.

To address these research gaps, this study focused on multi-ecoregion paddy fields in Liaoning Province, with fixed-point field monitoring experiments performed using a single rice cultivar. Liaoning Province is one of the main rice-producing regions of China. In 2025, its rice planting area reached 500.2 thousand hectares, with a total rice output of 4.061 million metric tons, highlighting its important position in national japonica rice production and food security. By integrating in situ monitoring data of climatic factors (mean temperature, precipitation, relative humidity, soil temperature, sunshine duration, etc.) and soil chemical parameters (pH, available nutrients, heavy metal content, mineral element concentrations, etc.), combined with untargeted metabolomics and multivariate statistical analysis, we aimed to dissect the differential accumulation patterns of rice metabolites and identify key regulatory factors under climate–soil synergy. This study will not only provide novel evidence for elucidating the effects of environmental factors on crops but also offer theoretical support for regional selection and breeding of high-quality rice varieties, precise regulation of rice quality formation, and food security assurance under climate change.

## 2. Materials and Methods

### 2.1. Fixed-Point Experimental Design and Sample Collection

**Figure 1:**
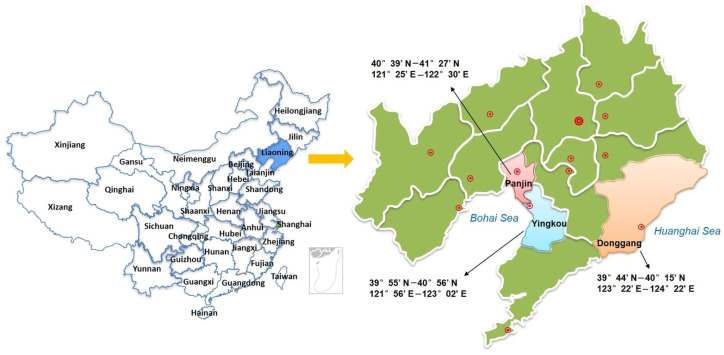
Implementation of fixed-point experiments and sample collection regions.

The fixed-point experimental bases were established in Panjin City (PJ), Yingkou City (YK), and Donggang City (DG) of Liaoning Province. These three regions, being coastal with abundant water resources, are key rice cultivation areas in Liaoning Province. Specifically, PJ and YK are located on the opposite banks of the Bohai Sea Basin, while DG is adjacent to the Yellow Sea Basin (Figure 1). In each rice planting region, three representative sampling points were selected. For each sampling plot, five subsamples were randomly collected using the five-point sampling method and mixed to form a representative composite sample. Each plot was continuously monitored for three consecutive years. A total of 27 rice composite samples were collected, including 9 from PJ, 9 from DG, and 9 from YK, with detailed sample information provided in Table S1. Rice cultivar Yanfeng 47 was uniformly planted and sampled across all three regions to eliminate confounding interference caused by genetic differences. Additionally, corresponding soil samples were collected from the subsurface position associated with each rice sample.

### 2.2. Analysis of Soil Multi-Elements in Different Rice Cultivation Regions

Each rice grain sample’s corresponding rhizosphere soil at a depth of 5 cm below the surface was collected and air-dried at room temperature, ground into powder, and then sieved through a 0.149 mm sieve. Approximately 0.2 g of soil powder was weighed into a pre-treated 60 mL microwave digestion vessel, followed by the addition of 5.0 mL HNO_3_ (Merck KGaA, Darmstadt, Germany), 4 mL HF (Thermo Fisher Scientific, Waltham, MA, USA), and 1 mL HClO_4_ (Thermo Fisher Scientific, Waltham, MA, USA). After soaking overnight, the mixture was heated in an electrothermal digestion system (ST36-iTOUCH, LabTech, Boston, MA, USA) under controlled temperature: heated at 100–110 °C for 120 min, and then ramped to 150–160 °C until white fumes evolved (about 90 min). The solution was checked for black particles; if abundant residues remained, an additional 1–2 mL HF was supplemented. For clear, colorless or pale yellow solutions with no visible residue, the temperature was increased to 180 °C for further evaporation until the acid volume was reduced to ~0.5 mL. The sample tube was then removed and cooled to room temperature. The cooled digest was then diluted to 25.0 mL with deionized water, thoroughly mixed, and filtered using a 0.45 μm aqueous membrane. A total of 25 elements (Mn, Zn, Cu, P, K, Fe, Mg, Ca, Cd, As, Se, Pb, Al, Sr, Rb, V, Co, Ni, Ga, Cs, Ag, Cr, U, Be and Ba) were determined by ICP-MS (Agilent 7900, Santa Clara, CA, USA) and ICP-OES (Agilent 5800, Santa Clara, CA, USA). The certified multi-element standard reference material for soil (GBW074473) was obtained from the Institute of Geophysical and Geochemical Exploration (IGGE, Tianjin, China). Detailed information on the instruments and reagents used in this experiment is available in previously published literature [14].

### 2.3. Analysis of Soil PH and Organic Matter in Different Rice Cultivation Regions

Determination of pH: Soil pH was determined via potentiometry using water as the extracting solution. Water was added to a flask to no more than two-thirds of its volume, boiled for 10 min and cooled. The flask was then sealed with a rubber stopper equipped with a soda-lime drying tube to prepare carbon dioxide-free water for subsequent experiments. Weigh 10.0 g ± 0.1 g of air-dried soil sample into a 50 mL tall beaker and add 25 mL of water. Seal the container, and then shake or stir vigorously for 5 min using a shaker or stirrer. After standing for 2 h, measure the pH with a calibrated pH meter (ORION STAR A211, Thermo Fisher Scientific, Waltham, MA, USA).

Determination of organic matter: Accurately weigh 0.5 g of air-dried sample sieved through a 0.25 mm sieve (accurate to 0.0001 g) into a hard test tube. Heat the sample and oxidize soil organic carbon with 10.00 mL of 0.4 mol/L potassium dichromate-sulfuric acid solution. Titrate the excess potassium dichromate with standard ferrous sulfate solution (0.1 mol/L). Calculate the organic carbon content from the consumed potassium dichromate using an oxidation correction coefficient, and then multiply by the constant 1.724 to obtain the soil organic matter content. For detailed procedures, refer to the Agricultural Industry Standard of the People’s Republic of China NY/T 1121.6-2006 [15].

### 2.4. Field Meteorological Data Collection in the Area Where Rice Samples Are Located

Meteorological data for the sampling sites were collected from a field weather station, covering the entire rice growth period from May to October each year. The meteorological data were recorded continuously for three years, corresponding to the years of sample collection. The weather stations are located in the prefecture-level city administrating the township where each sampling site is situated, and the actual distance between each sampling site and its corresponding weather station is less than 15 km.

### 2.5. Metabolomic Analysis of Rice

A 100 mg aliquot of liquid nitrogen-ground tissue sample was placed into an EP tube. At 4 °C, 500 μL of pre-chilled 80% methanol (Thermo Fisher, USA) was added, followed by vortex-mixing and incubation on ice for 5 min. The mixture was centrifuged at 15,000× *g* and 4 °C for 20 min. An appropriate volume of supernatant was transferred to a new EP tube, diluted with mass spectrometry-grade water to a final methanol concentration of 53%, vortex-mixed, and centrifuged again at 15,000× *g* and 4 °C for 20 min. The supernatant was collected and subjected to metabolomic profiling using liquid chromatography coupled with Orbitrap mass spectrometry (LC-Orbitrap MS) (Q Exactive™ HF/Q Exactive™ HF-X, Thermo Fisher, USA). An equal-volume aliquot from each experimental sample was pooled to prepare a quality control (QC) sample. A blank sample consisting of 53% methanol aqueous solution was also prepared, with the pretreatment procedure identical to that of the experimental samples [16].

Chromatographic conditions for the experiment: The column used was a Hypersil Gold column (C18) (Thermo Fisher Scientific, Waltham, MA, USA), with a column temperature of 40 °C and a flow rate set at 0.2 mL/min. In positive mode, mobile phase A consisted of 0.1% formic acid (Thermo Fisher, USA) and mobile phase B was methanol; in negative mode, mobile phase A was 5 mM ammonium acetate with the pH adjusted to 9.0 (Thermo Fisher, USA) and mobile phase B was methanol. The chromatographic gradient elution program is shown in Table S2. Mass Spectrometry Conditions: The scan range was set to *m*/*z* 100–1500. The ESI source settings were as follows: spray voltage: 3.5 kV, sheath gas flow rate: 35 psi, aux gas flow rate: 10 L/min, capillary temp: 320 °C, S-lens RF level: 60, aux gas heater temp: 350 °C, and polarity: set to positive ion mode and negative ion mode. MS/MS secondary scanning was performed as data-dependent scans [17].

The raw files obtained from mass spectrometry were imported into Compound Discoverer 3.3 software (hereafter referred to as CD 3.3) for spectral processing and database searching (against the MassList primary database and high-resolution secondary spectral databases mzCloud and mzVault), to obtain qualitative and quantitative results of metabolites. Subsequently, quality control (QC) was performed to ensure data accuracy and reliability: background ions were removed using the blank sample; original quantitative values were normalized via the formula sample original quantitative value (sum of sample metabolite quantitative values/sum of QC sample metabolite quantitative values) to generate relative peak areas; compounds with a coefficient of variation (CV) of relative peak areas exceeding 30% in QC samples were excluded. Finally, the identification and relative quantification results of metabolites were obtained. Identified metabolites were annotated using the KEGG database (https://www.genome.jp/kegg/pathway.html, accessed on 5 July 2025), HMDB database (https://hmdb.ca/metabolites, accessed on 22 July 2025), and LIPIDMaps database (http://www.lipidmaps.org/, accessed on 23 July 2025). All annotated metabolites were assigned to MSI Level 2 identification, which is defined as tentative identification based on matching experimental MS/MS fragmentation patterns against reference spectral libraries without the use of authentic chemical standards.

### 2.6. Data Analysis

Data analysis and visualization (including heatmaps and correlation plots) were performed using Origin 2021, with metabolite data standardized by z-score normalization. PLS-DA, together with its corresponding cross-validation and permutation test, was performed using MetaboAnalyst 6.0 online software (https://www.metaboanalyst.ca/, accessed on 16 September 2025). Spearman correlation coefficients and Mantel tests were calculated and visualized in R using the linkET, tidyverse, ggplot2, and veganpackages. Mantel tests were performed with 999 permutations.

## 3. Results

### 3.1. Analysis of Environmental Factors in Different Rice Cultivation Regions

#### 3.1.1. Basic Climatic Environment of Different Rice Cultivation Regions

The basic climatic conditions of the test rice cultivation regions (Panjin City, Yingkou City, and Donggang City), continuously monitored during the rice growing season, are shown in Table 1. Significant differences in average relative humidity, precipitation, and wind-related factors were observed among different production areas.

#### 3.1.2. Analysis of Basic Soil Chemical Environment in Different Rice Cultivation Regions

The basic soil conditions of the test rice cultivation regions (PJ, YK, and DG) are shown in Table 2. As shown in Table 2, significant differences were observed in the pH and the contents of elements Ca, Se, U and Sr in the paddy soils of the three main cultivation regions. Specifically, when ranked in descending order of the contents of Ca and Sr elements and pH, the three regions all followed the order PJ > YK > DG; when ranked in descending order of Se and U content, the order was DG > YK > PJ. The organic matter content in the paddy soil of DG was significantly higher than that in PJ and YK.

### 3.2. Metabolite Composition of Rice Under Different Production Regions

#### 3.2.1. Identification and Annotation of Metabolites

Through spectral database searching and molecular formula prediction of molecular ion peaks and fragment ions [18], 869 and 706 metabolites were identified in the test rice samples under positive and negative ion modes, respectively. Classification of metabolites revealed that the top six metabolite classes in both positive and negative ion modes were consistently lipids and lipid-like molecules, organic acids and derivatives, organoheterocyclic compounds, phenylpropanoids and polyketides, benzenoids, and organic oxygen compounds. Further annotation of the identified metabolites in rice samples was performed using the KEGG, HMDB, and LIPID MAPS databases to characterize their pathways and classifications. Based on the KEGG database, a total of 502 metabolites were annotated, mainly involving three pathways: environmental information processing, genetic information processing, and metabolism. Using the HMDB database, 811 metabolites were annotated, with lipids and lipid-like molecules being the most abundant. With the LIPID MAPS database, 269 metabolites were annotated, primarily classified into 7 major categories: Fatty Acyls, Glycerolipids, Glycerophospholipids, Polyketides, Prenol Lipids, Sphingolipids, and Sterol Lipids (Figure S1).

#### 3.2.2. Screening and Analysis of Differential Metabolites

**Figure 2:**
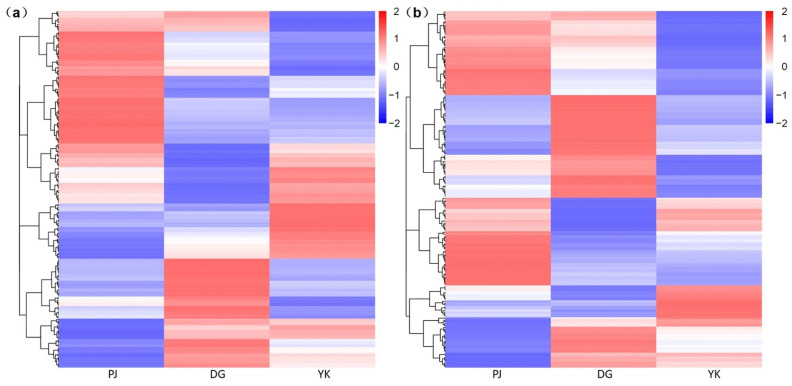
Heatmap of differential metabolites in rice from three major cultivation regions. (**a**) Heatmap of differential metabolites in positive ion mode; (**b**) heatmap of differential metabolites in negative ion mode. PJ stands for Panjin, DG stands for Donggang, and YK stands for Yingkou.

**Figure 3:**
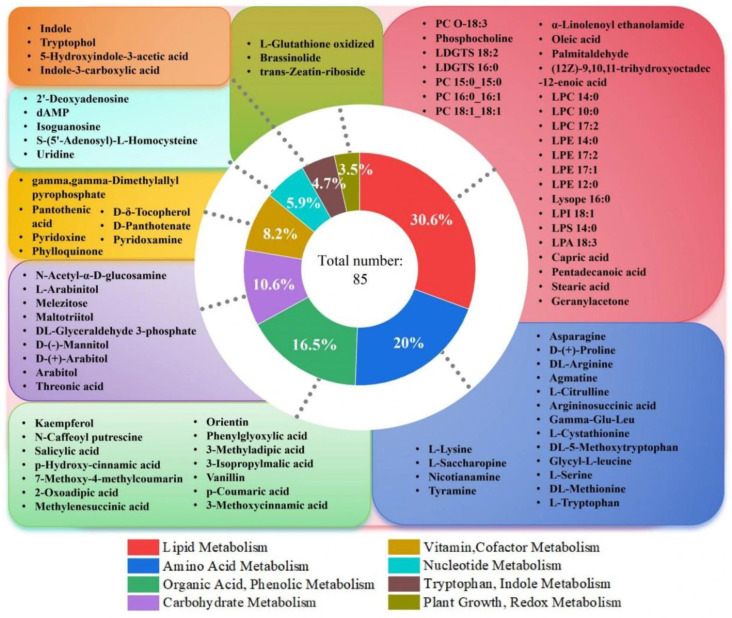
Metabolites with inter-group differences exist in rice grown in three distinct planting environments.

To comprehensively evaluate the metabolite differences in rice produced from three major rice cultivation regions, hierarchical clustering analysis (HCA) was performed on all differential metabolites obtained from each pairwise comparison. The relative quantitative values of differential metabolites were normalized, transformed, and clustered, generating a simplified heatmap (Figure 2) and a detailed heatmap (provided in Figures S2 and S3). Through literature verification, 85 inter-group differential metabolites with a high likelihood of presence in rice were screened, including 49 in positive ion mode and 36 in negative ion mode. A total of 8 metabolic pathways were classified, including 26 metabolites involved in lipid metabolism, 17 in amino acid metabolism, 14 in organic acid and phenolic metabolism, 9 in carbohydrate metabolism, 7 in vitamin and cofactor metabolism, 5 in nucleotide metabolism, 4 in tryptophan and indole metabolism, as well as 3 in plant growth and redox metabolism (Figure 3). Overall, the metabolic profiles of rice from the three regions exhibited significant spatial heterogeneity, indicating that the composition of rice metabolites is largely influenced by growth environments.

#### 3.2.3. Mining of Characteristic Metabolites for Rice from Different Geographical Origins

**Figure 4:**
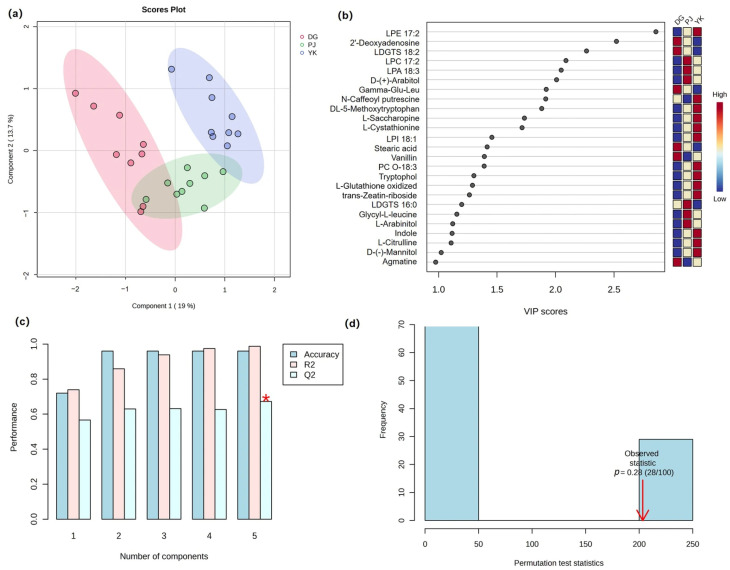
PLS-DA model established based on 85 screened differential metabolites. (**a**) Scores plot based on PLS-DA analysis, (**b**) VIP diagram established based on PLS-DA analysis, (**c**) the corresponding cross-validation test plot of (**a**), (**d**): the results of 100 permutation tests of (**a**); PJ stands for Panjin, DG stands for Donggang, and YK stands for Yingkou. * indicates the optimal number of model components with the maximum Q^2^ value.

To explore the effectiveness of using metabolites for traceability of rice from geographically proximate regions and to identify characteristic marker metabolites contributing most to group differentiation, we further screened 85 metabolites with significant differences between groups based on the previous hierarchical clustering analysis, and used the chemometric analysis method (PLS-DA) to group the rice samples from the three regions into geographical source groups (Figure 4). Figure 4a showed the scores plot established based on PLS-DA analysis, indicating that the model built using these 85 differential metabolites can essentially achieve separation of rice from three distinct environmental cultivation regions. As illustrated in the variable importance in projection (VIP) plot (Figure 4b), among the 85 metabolites, 24 metabolites with a VIP value greater than 1.0 contributed greatly to the group separation. Metabolites with higher accumulation in rice from the PJ region were lysophosphatidylcholine (LPC) 17:2, lysophosphatidic acid (LPA) 18:3, D-(+)-Arabitol, lysodiacylglyceryltrimethylhomoserine (LDGTS) 16:0, Glycyl-L-leucine and L-Arabinitol, which were primarily associated with three primary metabolic pathways: lipid metabolism, amino acid metabolism, and carbohydrate metabolism. For rice samples from the DG region, the highly accumulated metabolites consisted of 2′-Deoxyadenosine, LDGTS 18:2, Gamma-Glu-Leu, stearic acid and vanillin, mainly involving primary metabolism, including nucleotide metabolism, lipid metabolism and amino acid metabolism, as well as secondary metabolic pathways of organic acid and phenolic metabolism. In rice from the YK region, metabolites with relatively higher contents were lysophosphatidylethanolamine (LPE) 17:2, N-Caffeoyl putrescine, DL-5-Methoxytryptophan, L-Saccharopine, L-Cystathionine, Lysophosphatidylinositol (LPI) 18:1, O-alkyl phosphatidylcholine (PC O)-18:3, Tryptophol, L-Glutathione oxidized, trans-Zeatin-riboside, Indole, L-Citrulline and D-(-)-Mannitol. These metabolites were mainly involved in three primary metabolic pathways (lipid metabolism, carbohydrate metabolism and amino acid metabolism), together with multiple secondary metabolic pathways, including tryptophan and indole metabolism, organic acid and phenolic metabolism, as well as plant growth and redox metabolism.

Generally, parameters with VIP > 2.0 are identified as the most significant discriminant factors [19]. To improve the accuracy of regional characteristic markers, we screened characteristic marker metabolites for PJ, DD, and YK rice from the metabolites with VIP > 2.0. Consequently, LPC 17:2, LPA 18:3, and D-(+)-Arabitol were identified as characteristic marker metabolites for rice from PJ; 2′-Deoxyadenosine and LDGTS 18:2 for rice from DG; and LPE 17:2 for rice from YK. Cross-validation results showed that R^2^ was close to 1.0 and Q^2^ > 0.6, indicating that the grouping model had good data interpretability and predictive power (Figure 4c). However, 100 permutation tests revealed that 28 out of 100 random models outperformed the current model, suggesting that the grouping model based on these 85 metabolites cannot effectively discriminate the geographical origins of rice at a small geographic scale (Figure 4d). Nevertheless, the six characteristic metabolites with VIP > 2.0 can provide potential parameters for rice geographical traceability at a small scale. Overall, region-specific marker metabolites in rice from different regions were dominated by primary metabolites.

### 3.3. Correlation Analysis Between Climate Factors and the Metabolic Composition of Rice

**Figure 5:**
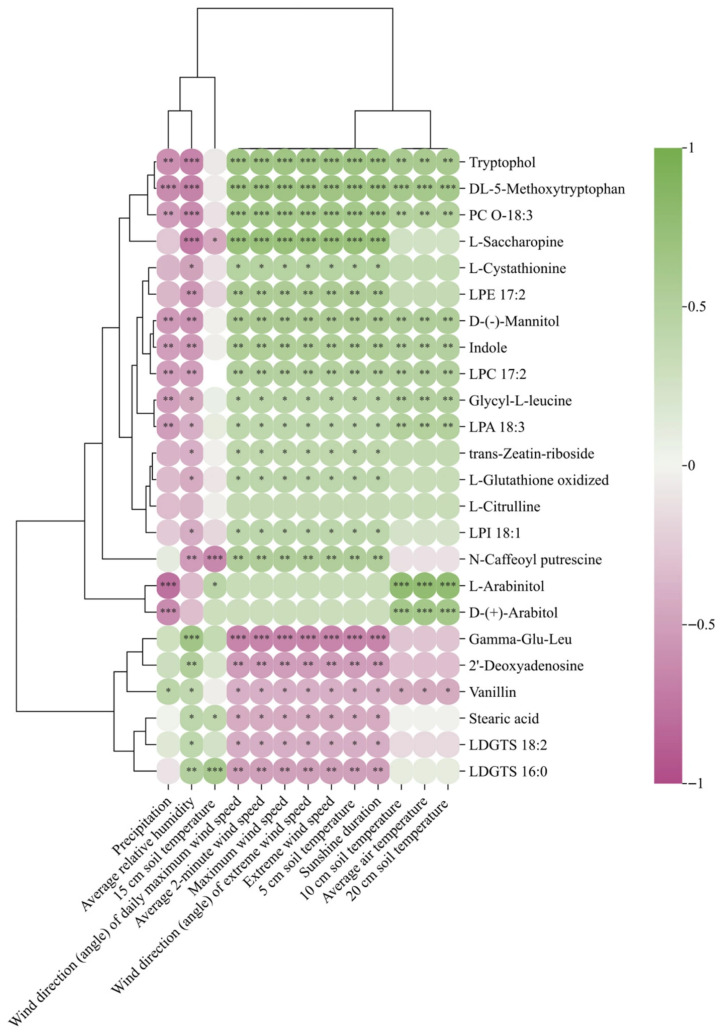
Correlation analysis between metabolite composition in rice and climate and environmental factors. Note: * *p* < 0.05, significant difference; ** *p* < 0.01, highly significant difference; *** *p* < 0.001, extremely significant difference. Green indicates positive correlation coefficients, whereas pink represents negative correlation coefficients.

To elucidate the intrinsic correlations between climatic environmental factors and the accumulation of region-specific characteristic metabolites in rice grains, a correlation heatmap combined with cluster analysis was constructed in this study based on 24 key metabolites (VIP > 1.0) with great contribution to the discrimination of rice samples from three cultivated regions, as well as corresponding meteorological datasets (Figure 5). Overall, distinct clustered correlation patterns were observed between all detected environmental variables and the 24 pivotal differential metabolites. Collectively, sunshine duration, average relative humidity and wind regime were identified as the dominant climatic drivers governing the metabolite profiling of rice grains. Meanwhile, characteristic metabolites derived from different producing regions exhibited distinctly divergent response patterns to climatic factors.

According to the correlation patterns, all metabolites were classified into three subclusters, and metabolites within the same cluster branch presented analogous response trends to climatic factors. Specifically, LPE 17:2, the marker metabolite enriched in the YK region, was clustered in the first branch, which exhibited significantly strong positive correlations with 5 cm soil temperature, sunshine duration and various wind-related meteorological factors, whereas it displayed extremely strong negative correlations with precipitation and average relative humidity. On the contrary, the high-abundance signature metabolites from the DG region (2′-Deoxyadenosine, LDGTS 18:2) displayed completely opposite correlation tendencies. These metabolites were positively correlated with average relative humidity, yet negatively correlated with sunshine duration, 5 cm soil temperature, and wind-related climatic variables, including wind speed and wind direction. The predominant differential metabolites in the PJ region (LPC 17:2, LPA 18:3, D-(+)-Arabitol) belonged to the same first cluster branch, and all of them exhibited markedly strong positive correlations with multi-layer soil temperature (10 cm, 20 cm) and ambient average air temperature, alongside extremely strong negative correlations with precipitation. In summary, characteristic metabolites of rice from YK, DG and PJ regions presented region-specific correlation patterns with corresponding local environmental factors, including soil temperature, sunshine duration, humidity and wind conditions. The unique integrated environmental attributes of each producing region, comprising hydrothermal conditions, soil properties and wind regimes, collectively determined the differentiated accumulation profiles of region-specific metabolites in rice grains.

### 3.4. Correlation Analysis Between Soil Environmental Factors and Rice Metabolite Composition

**Figure 6:**
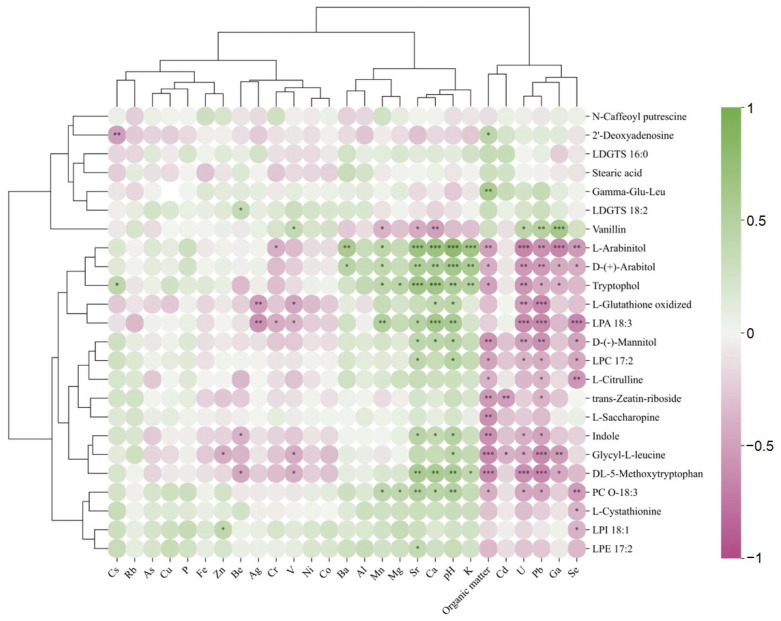
Correlation analysis between metabolite composition in rice and soil chemical factors. Note: * *p* < 0.05, significant difference; ** *p* < 0.01, highly significant difference; *** *p* < 0.001, extremely significant difference. Green indicates positive correlation coefficients, whereas pink represents negative correlation coefficients.

To clarify the driving effects of soil physicochemical properties on the differential accumulation of characteristic metabolites in rice grains, a correlation cluster heatmap analysis was performed between all marker metabolites and soil environmental factors, including mineral element contents, pH, organic matter and heavy metal contents, with results illustrated in Figure 6. Hierarchical clustering results revealed that all differential metabolites were divided into two major clusters according to their environmental response characteristics, and metabolites within each cluster further formed distinct subclasses. Overall, soil pH, organic matter, and elements, including Sr, Ca, U, Pb and Se, were closely associated with the contents of most metabolites.

Specific correlation analysis indicated that region-specific marker metabolites from different producing regions exhibited distinctly differentiated response patterns to soil environmental factors. Among them, the marker metabolites enriched in the YK region and the dominant differential metabolites in the PJ region were grouped into the same major cluster, yet belonged to separate subclasses. Specifically, LPE 17:2, the characteristic metabolite of the YK region, was significantly positively correlated with soil Sr content. The dominant marker metabolites in the PJ region (LPC 17:2, LPA 18:3, D-(+)-Arabitol) showed positive correlations with multiple soil mineral elements (Ca, Sr, Mn, K, etc.), soil pH and fundamental physicochemical indices, with numerous correlations reaching significant levels, whereas they were negatively correlated with the contents of organic matter, U, Pb and Se. By comparison, the high-abundance characteristic metabolites from the DG region, which is geographically distant from YK and PJ regions (2′-Deoxyadenosine, LDGTS 18:2), were clustered into the other major metabolite cluster. These metabolites presented negative correlations with soil organic matter. In addition, 2′-Deoxyadenosine showed a negative correlation with Cs, while LDGTS 18:2 was positively correlated with Be.

### 3.5. Analysis of Interactive Relationships Between Climate, Soil Chemical Environment and Rice Metabolites

**Figure 7:**
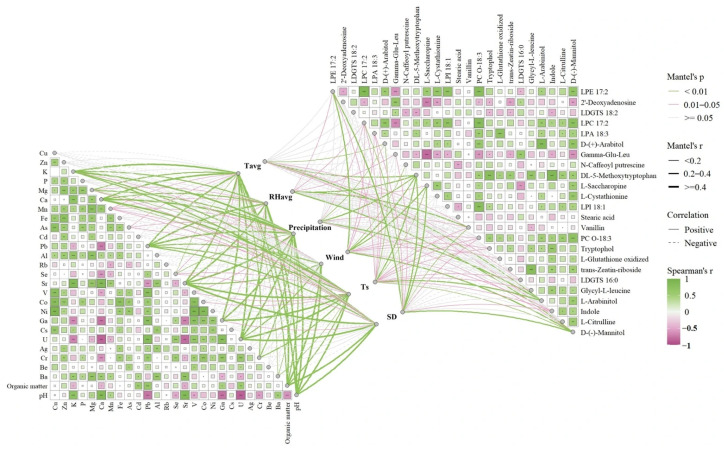
Interactive relationships between climate, soil chemical environment and rice metabolites. Note: Tavg, RHavg, Precipitation, SD represent average air temperature, average relative humidity, precipitation, sunshine duration, respectively. Wind represents comprehensive wind characteristics involving average 2 min wind speed, wind direction (angle) of daily maximum wind speed, maximum wind speed, wind direction (angle) of extreme wind speed, extreme wind speed. Ts represents multi-layer soil temperature, including 5 cm soil temperature, 10 cm soil temperature, 15 cm soil temperature and 20 cm soil temperature. Mantel tests were performed with 999 permutations. Mantel’s *p*-value indicates statistical significance, which is distinguished by line colors: green lines indicate extremely significant correlations (*p* < 0.01); pink lines indicate significant correlations (0.01 < *p* < 0.05); gray lines denote non-significant correlations (*p* > 0.05). Line thickness represents Mantel’s r-value (correlation coefficient), which reflects the strength and direction of the correlation between two matrices and ranges from −1 to 1. An r value greater than 0 indicates a positive correlation, an r value less than 0 indicates a negative correlation, and r = 0 implies no correlation. In terms of correlation magnitude: (|r| < 0.2) refers to a weak correlation, (0.2 ≤ |r| < 0.4) refers to a moderate correlation, and (|r| ≥ 0.4) refers to a strong correlation. Solid lines represent positive correlations, and dashed lines represent negative correlations. For the heatmap based on Spearman’s correlation analysis, green indicates positive correlations and pink indicates negative correlations. * *p* < 0.05, significant difference; ** *p* < 0.01, highly significant difference; *** *p* < 0.001, extremely significant difference.

To systematically elucidate the holistic correlations and multi-dimensional interactive mechanisms among soil physicochemical properties, regional climatic factors, and differential metabolites in rice grains, Spearman correlation analysis and Mantel test were integrated in this study to construct a multi-environment factor–metabolite correlation network, as illustrated in Figure 7. According to the internal correlation characteristics of each group derived from Spearman analysis, the heatmap of soil physicochemical indices on the left revealed extensive significant pairwise correlations among multiple mineral elements, pH, organic matter and other soil parameters, indicating intensive internal synergistic and antagonistic effects among soil physicochemical properties in the study area. As shown in the metabolite correlation heatmap on the right, characteristic metabolites from identical producing regions presented highly positively correlated aggregation. Specifically, region-specific metabolite clusters were independently formed, including LPE 17:2 (characteristic metabolite of YK rice), high-abundance metabolites from the DG region (2′-Deoxyadenosine, LDGTS 18:2), and dominant differential metabolites from the PJ region (LPC 17:2, LPA 18:3, D-(+)-Arabitol). This result further verified the validity of these metabolites as regional characteristic biomarkers.

Intergroup correlation analysis based on the Mantel test demonstrated extremely strong, significant positive overall correlations among climatic factors, soil physicochemical properties and rice metabolite profiles. All core meteorological factors were closely linked to the soil environmental system via thick solid green lines, with extremely significant correlations (*p* < 0.01) and high correlation coefficients (r ≥ 0.4), confirming prominent synergistic interactions between regional meteorological conditions and soil physicochemical properties. Meanwhile, all six climatic factors exhibited extremely significant and strong positive correlations with the overall rice metabolite community. Among them, integrated wind condition, soil temperature (Ts) and sunshine duration (SD) were identified as the core meteorological driving nodes with the highest correlation intensity.

Focusing on the response patterns of region-specific marker metabolites from key producing areas, further analysis revealed that LPE 17:2, the core marker metabolite enriched in the YK rice-producing region, exhibited extremely significant, strong positive correlations with integrated wind condition and sunshine duration, while showing negative correlations with precipitation and relative air humidity. Meanwhile, it was significantly positively correlated with soil organic matter, pH, and the contents of Sr, U and Pb elements, which partially corroborated the previous correlation analysis results. High-abundance characteristic metabolites in the DG region (2′-Deoxyadenosine, LDGTS 18:2) formed an independent metabolite cluster, and their correlations with climatic conditions failed to reach statistical significance. Dominant differential metabolites of the PJ region (LPC 17:2, LPA 18:3, D-(+)-Arabitol) clustered into the same metabolite subgroup. Among them, phospholipid metabolites LPC 17:2 and LPA 18:3 displayed moderately significant positive correlations with integrated wind condition, sunshine duration and soil temperature, and strong significant positive correlations with soil organic matter, pH, and multiple soil mineral elements, including Sr, U, Pb, K and Ca.

Collectively, the multi-dimensional correlation results fully verified that regional climate and soil physicochemical properties synergistically interact to jointly drive the differential accumulation and spatial differentiation of characteristic metabolites in rice from diverse producing areas. Meteorological factors dominated by sunshine duration, wind condition and soil temperature, together with soil chemical factors, including soil organic matter, pH, and elements such as K, Ca, Pb, Sr and U, constitute the core driving factors underlying the spatial differentiation of region-specific rice metabolites.

## 4. Discussion

Untargeted metabolomic analysis revealed that the metabolites identified in the tested rice samples were classified into six core categories, including lipids, organic acids, and phenylpropanoids. This classification result was consistent with the major metabolite profiles of rice reported in previous studies [20,21]. Hierarchical cluster analysis (HCA) demonstrated remarkable spatial heterogeneity in the metabolic profiles of rice from three primary producing regions. PLS-DA was performed on 85 authentic differential metabolites screened from rice samples. Although the established model required further optimization, it effectively distinguished rice samples from YK, geographically adjacent producing areas. These findings highlighted the potential value of combined multi-metabolite signatures for traceability of rice origins in small-scale adjacent production regions.

Among these metabolites, 24 metabolites with VIP > 1.0 exhibited prominent contributions to geographical origin classification, which were mainly involved in eight metabolic pathways, including lipids, amino acids, phenolic acids, and plant hormones. These pathways were highly consistent with those previously reported to be closely associated with geographical traits in untargeted metabolomic traceability studies of rice from Heilongjiang and Jiangsu Provinces [12]. The PJ region is a typical saline-alkali soil cultivation area. Rice produced in this area was enriched with LPC 17:2, LPA 18:3, and sugar alcohols (D-(+)-Arabitol, L-Arabinitol). Phospholipids are vital components of cell membranes and exert multifunctional roles in regulating ion channels and nutrient uptake [22]. As a pentahydric alcohol, Arabitol is commonly detected in drought-tolerant organisms such as lichens and plays a crucial role in osmotic adjustment [23]. Accordingly, our results verified that the distinctive saline-alkali soil environment in this region altered phospholipid metabolism and the accumulation of osmotic adjustment substances. Compared with PJ and YK adjacent to the Bohai Sea, the DG region features a unique mild and humid microclimate formed by the convergence of maritime air masses and cold humid mountainous air currents owing to its proximity to the Yellow Sea. High ambient humidity is widely recognized as a key environmental factor triggering the prevalence of field crop diseases [24], and rice diseases have long occurred frequently in this region. The activation of systematic defense responses is speculated to be primarily responsible for the elevated contents of secondary metabolites, including 2′-Deoxyadenosine, stearic acid, and vanillin, in DG rice samples. Relative to PJ and DG, rice from the YK region enjoys relatively low market popularity, accompanied by extensive, diversified field management practices with insufficient specialization and standardization. The significant accumulation of multiple amino acid derivatives (e.g., L-Cystathionine, L-Citrulline), cytokinins (trans-zeatin riboside), and indole compounds implied enhanced nitrogen availability or specific agrochemical application in the local cultivation environment, which stimulated the activation of nitrogen metabolism and hormone biosynthesis pathways. In addition, lipids, including LPE, LPC, and LPI, displayed differential accumulation patterns across all regions, confirming the high sensitivity of lipid metabolism to environmental factors such as ambient temperature and cultivation practices [25,26]. Furthermore, the regional variations in phenylpropanoid derivatives (e.g., N-caffeoylputrescine, vanillin) further supported the fundamental role of plant secondary metabolites in plant growth, development and environmental adaptation [27].

According to the rigorous screening criterion of VIP > 2.0, region-specific characteristic marker metabolites for each producing area were further identified. Specifically, the characteristic markers of PJ rice were LPC 17:2, LPA 18:3 and D-(+)-Arabitol, those of DG rice were 2′-Deoxyadenosine and LDGTS 18:2, and LPE 17:2 was identified as the distinctive metabolite of YK rice. Notably, the most representative characteristic metabolites from all three regions were all categorized as lipids. Previous studies have demonstrated that lipid metabolites are essential components of biological membranes. Beyond structural support in plants, they actively participate in signal transduction and metabolic regulation under both abiotic and biotic stress conditions. Accordingly, their contents and compositional profiles are inevitably modulated by environmental factors such as temperature and light, when plants adopt lipid-associated strategies to adapt to nutrient deficiency, toxic stress and fluctuating environments [22]. The prominent geographical specificity of lipid metabolites observed in this study further verified their close correlation with environmental variables, indicating that such lipid metabolites can serve as core potential markers for rice geographical origin traceability at a small geographical scale. Recent studies have demonstrated that at the municipal traceability scale, differential metabolites among sample groups are mainly categorized into lipids, organic acids and their derivatives, amino acids and their derivatives, benzene and substituted derivatives, as well as saccharides and sugar alcohols. Among them, variations in lipid metabolism serve as the most critical discriminatory signal, especially membrane-associated lipids such as LysoPG, PA, PE and PC characteristic compounds [28]. Our results are highly consistent with this conclusion.

In addition, 2′-Deoxyadenosine (nucleotide metabolite) from the DG region and D-(+)-Arabitol (carbohydrate metabolite) from the PJ region further expanded the metabolite marker library for rice origin traceability, suggesting potential as traceability markers for traceability of rice originating from geographically adjacent regions. Overall, the exclusive geographical markers across the three regions were dominated by primary metabolites, including lipids, carbohydrates and nucleotides. Secondary metabolites mainly contributed to the differentiation of overall metabolic backgrounds among regions rather than serving as core traceability markers. This phenomenon reflected that the primary basal metabolism in rice grains exhibits higher sensitivity to habitat and geographical variations in metabolic regulation.

Combined with the correlation analysis of climatic and environmental factors, sunshine duration, relative air humidity and regional wind regime were identified as the core meteorological variables closely associated with the differentiation of overall rice metabolic profiles. Meanwhile, characteristic metabolites from different producing regions exhibited distinctly divergent responses to environmental factors. The marker metabolites of YK rice displayed significantly positive correlations with sunshine duration, soil temperature and wind speed, while showing significantly negative correlations with precipitation and air humidity. In contrast, characteristic metabolites from the DG region presented an opposite environmental response pattern, preferring environments with high humidity and weak light intensity. By comparison, the marker metabolites in PJ rice were primarily regulated by multi-layer soil temperature and regional average temperature, accompanied by obvious negative responses to precipitation. These differential response patterns indicated that subtle variations in hydrothermal conditions and wind environments within geographically adjacent areas could drive the synthesis and accumulation of lipids and sugar alcohols, thereby forming unique metabolic fingerprints for each producing region.

Soil physicochemical properties also exerted remarkable effects on the spatial differentiation of rice metabolites, and the responses of regional marker metabolites to soil elements differed markedly. Although characteristic metabolites of rice from geographically adjacent YK and PJ were clustered into one major category, they were subdivided into distinct subclusters. Specifically, metabolites from YK were mainly responsive to soil Sr content. A series of markers in PJ rice presented significantly positive correlations with soil pH, Ca, Sr, Mn, K and other mineral nutrients, and negative correlations with soil organic matter and heavy metal elements. In contrast, metabolites from the relatively distant DG region formed an independent cluster, which were generally positively correlated with soil organic matter and displayed unique specific responses to trace elements. Collectively, Sr and soil organic matter played pivotal roles in the differential accumulation of rice metabolites induced by small-scale environmental heterogeneity. In particular, Sr may serve as a candidate marker pending validation for metabolic differentiation among extremely adjacent geographical producing areas. Accordingly, both factors possess promising research value and application potential in subsequent studies on rice origin traceability and habitat response. In summary, soil nutrient composition, mineral element background and soil acid–base properties directly regulated grain metabolic synthesis, and jointly shaped the geographical specificity of rice metabolic components together with regional climatic conditions.

Multi-factor interaction analysis was further performed via Spearman correlation analysis combined with the Mantel test to clarify the multi-dimensional interactive relationships among climate, soil environment and rice metabolites. Strong synergistic interactions were observed between climatic factors and soil physicochemical properties across the study area. Meteorological conditions indirectly affected grain metabolic processes by regulating soil hydrothermal status and nutrient availability. Extremely significant overall correlations were detected between the entire environmental system and rice metabolic profiles, among which sunshine duration, soil layer temperature and comprehensive wind regime were the dominant driving factors. The multi-environmental response patterns of core markers in each region were consistent with single-factor correlation results. For instance, LPE 17:2, the characteristic marker of YK rice, was jointly affected by sunshine duration, wind regime and soil Sr. Phospholipid metabolites (LPC 17:2, LPA 18:3) and sugar alcohol metabolites in the PJ region coordinately responded to thermal meteorological factors and multiple soil mineral elements. Metabolites from the DG region exhibited relatively weak overall environmental correlations, and their metabolic phenotypes were predominantly governed by the distinctive regional humid climate and inherent soil properties. Integrated multi-dimensional results demonstrated that regional climate and soil environment did not independently affect metabolite accumulation in rice grains. Instead, their internal synergistic coupling effects jointly drove the differential enrichment of characteristic metabolites in rice from adjacent producing regions. Meteorological variables, including sunshine duration, soil temperature and wind regime, as well as soil properties involving pH, organic matter and mineral element composition, were critical environmental drivers responsible for the differentiation of metabolic fingerprints of rice at a small geographical scale. The screened characteristic metabolites in this study can provide metabolomic evidence for geographical origin traceability of rice from adjacent regions and mechanism analysis of regional quality formation.

Overall, this study clarifies the correlations between climate, soil and rice metabolism, and screens candidate region-specific metabolic markers. The findings can provide theoretical references for precise field water and nutrient management as well as rice quality improvement, and also offer strategies for small-scale rice geographical origin tracing and brand development of characteristic rice varieties. Most of the identified characteristic metabolites are functional lipids, together with D-(+)-Arabitol, with potential benefits for low-sugar diets. Unraveling their accumulation patterns in response to environmental factors facilitates the selection of suitable planting environments and the breeding of nutrient-enriched, high-quality rice to meet public dietary and health demands. Moreover, this research enriches the theory governing interactions between rice plants and their paddy field environments, and verifies that strontium and soil organic matter act as potential indicators for rice metabolic differentiation. It lays a foundation for farmland ecological zoning, soil fertility improvement and formulation of green fertilization regimes, thereby supporting the sustainable development of paddy ecosystems.

However, this work still has some limitations. First, the sample size across different producing regions was relatively small, which may reduce statistical representativeness and weaken the robustness and generalizability of our multi-source fusion traceability model. Second, sampling was only performed for fewer than three consecutive harvest years. Interannual variations in climate and field management alter rice elemental, isotopic and metabolomic profiles, so the screened discriminative markers cannot fully reflect long-term stable regional differences. Third, these candidate biomarkers were only verified via internal cross-validation; external validation with independent blind samples was not conducted, leaving their practical reliability for identifying unknown rice samples unconfirmed. Future work will expand the sampling scale, carry out long-term multi-year collection, and adopt blind sample validation to further improve the practicability of this origin identification system.

## 5. Conclusions

**Figure 8:**
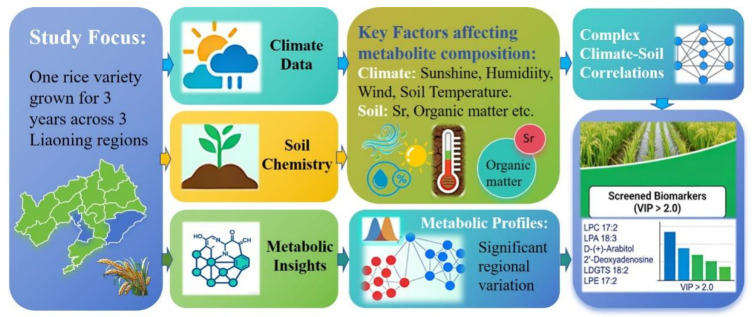
Simplified schematic diagram of research conclusions.

This study took rice from three major producing areas as the research object and adopted untargeted metabolomics combined with multi-dimensional analysis methods to explore the correlation and interaction rules between soil, climatic factors and rice metabolites, identify regional characteristic markers, and reveal the impact of environmental factors on the differentiation of rice metabolic profiles. The main conclusions are as follows (Figure 8): (1) The PLS-DA model constructed based on differential metabolites has potential for small-scale rice geographical origin traceability; with VIP > 2.0 as the screening threshold, the core characteristic markers of each producing area were determined: PJ is LPC 17:2, LPA 18:3, D-(+)-Arabitol, DG is 2′-Deoxyadenosine, LDGTS 18:2, and YK is LPE 17:2; the markers are mainly primary metabolites such as lipids. (2) Climatic factors significantly drive the differential accumulation of rice metabolites, with sunshine duration, relative humidity, wind regime and soil temperature as the main influencing factors; the markers of YK rice respond to long sunshine, high temperature and low humidity environments, DG is sensitive to high humidity and weak light, and PJ is positively regulated by air and soil temperature. (3) Soil physicochemical properties and mineral elements are important influencing factors for rice metabolic differentiation. The markers of PJ rice are positively correlated with soil mineral elements such as Ca, Sr and K, and negatively correlated with organic matter and heavy metals; the markers of YK rice have a prominent response to Sr elements; the markers of DG rice are independently clustered and positively correlated with organic matter; and Sr elements and organic matter have important indicative roles in small-scale rice metabolic differences. (4) Spearman correlation and Mantel test showed that there is a synergistic coupling effect between climate and soil environment, which jointly regulates the spatial differentiation of rice metabolites; sunshine duration, wind, soil temperature, soil pH and mineral element composition are the core driving factors for the differences in metabolic fingerprints of rice from adjacent producing areas. In summary, the findings of this study provide a metabolic theoretical basis for small-scale geographical origin traceability of rice and the elucidation of quality formation mechanisms in different producing regions. The established marker system has promising application potential in practical work such as rice quality control, geographical origin certification and origin adulteration detection. Further research will expand sampling areas to carry out multi-location validation experiments, so as to further verify the applicability of these markers for origin traceability over a larger geographical range.

## Figures and Tables

**Table 1:** Climatic environmental conditions of the test rice cultivation regions.

Climatic Factors (Units)	PJ	DG	YK
Average air temperature (°C)	21.69 ± 4.26	20.29 ± 4.55	20.96 ± 4.51
Average relative humidity (%) *	71.85 ± 11.58 b	79.94 ± 7.61 a	69.73 ± 11.53 b
Precipitation (mm) *	21.81 ± 25.82 c	33.84 ± 36.49 a	27.80 ± 28.38 b
Average 2 min wind speed (m/s) *	2.84 ± 0.67 b	1.96 ± 0.71 c	3.23 ± 0.85 a
Wind direction (angle) of daily maximum wind speed (m/s) *	185.95 ± 31.65 b	177.31 ± 29.49 c	210.39 ± 46.16 a
Maximum wind speed (m/s) *	6.00 ± 1.14 b	4.11 ± 1.55 c	6.71 ± 1.65 a
Wind direction of extreme wind speed (angle) *	190.36 ± 33.00 b	173.30 ± 30.75 c	210.98 ± 34.92 a
Extreme wind speed (m/s) *	9.75 ± 1.68 a	6.06 ± 2.39 b	9.91 ± 2.10 a
5 cm soil temperature (°C)	22.91 ± 5.14	22.88 ± 5.35	23.30 ± 5.41
10 cm soil temperature (°C)	23.12 ± 4.89	22.37 ± 5.17	23.03 ± 5.22
15 cm soil temperature (°C)	23.00 ± 4.71	22.17 ± 5.06	21.92 ± 4.77
20 cm soil temperature (°C)	22.58 ± 4.52	21.98 ± 4.93	22.55 ± 4.87
Sunshine duration (h)	7.41 ± 1.37	6.66 ± 1.51	7.95 ± 1.57

Note: PJ stands for Panjin, DG stands for Donggang, and YK stands for Yingkou. Asterisks (*) and letters indicate significant differences among groups (*p* < 0.05).

**Table 2:** Environmental conditions of paddy soils in different rice cultivation regions.

Soil Chemical Factors	PJ	DG	YK
pH *	7.54 ± 0.14 a	5.36 ± 0.14 c	6.88 ± 0.30 b
Organic matter (g/kg) *	35.10 ± 6.98 b	46.27 ± 2.87 a	30.83 ± 5.43 b
Cu (mg/kg)	23.89 ± 4.99	24.83 ± 3.01	26.87 ± 3.74
Zn (mg/kg)	82.33 ± 17.56	88.56 ± 8.43	89.39 ± 9.72
K (g/100 g)	2.31 ± 0.10	1.91 ± 0.23	2.11 ± 0.08
P (mg/kg)	659.74 ± 103.84	665.71 ± 202.16	695.17 ± 169.46
Mg (g/100 g)	0.77 ± 0.15	0.59 ± 0.16	0.73 ± 0.05
Ca (g/100 g) *	0.72 ± 0.21 a	0.18 ± 0.09 c	0.40 ± 0.13 b
Mn (mg/kg)	433.63 ± 90.14	308.99 ± 91.78	435.28 ± 99.27
Fe (g/100 g)	2.89 ± 0.45	3.17 ± 0.25	3.30 ± 0.32
As (mg/kg)	7.74 ± 3.85	7.28 ± 1.48	8.29 ± 1.89
Cd (mg/kg)	0.17 ± 0.06	0.18 ± 0.05	0.15 ± 0.04
Pb (mg/kg)	24.99 ± 3.06	32.06 ± 2.24	26.39 ± 2.98
Al (g/100 g)	4.94 ± 1.05	4.25 ± 0.569	4.45 ± 0.61
Rb (mg/kg)	59.16 ± 11.73	59.13 ± 8.38	57.22 ± 11.25
Se (mg/kg) *	0.08 ± 0.01 c	0.50 ± 0.13 a	0.38 ± 0.10 b
Sr (mg/kg) *	191.59 ± 15.97 a	113.89 ± 28.26 c	163.97 ± 9.32 b
V (mg/kg)	75.00 ± 10.09	88.76 ± 6.398	84.05 ± 12.27
Co (mg/kg)	11.70 ± 1.94	13.67 ± 0.81	13.80 ± 2.12
Ni (mg/kg)	31.10 ± 5.74	35.35 ± 3.35	35.78 ± 5.75
Ga (mg/kg)	14.96 ± 1.33	18.51 ± 0.62	17.64 ± 1.36
Cs (mg/kg)	5.31 ± 0.76	4.80 ± 0.50	5.29 ± 1.06
U (mg/kg) *	2.52 ± 0.22 c	4.71 ± 0.54 a	3.15 ± 0.58 b
Ag (mg/kg)	0.10 ± 0.01	0.11 ± 0.03	0.09 ± 0.03
Cr (mg/kg)	41.65 ± 10.41	63.16 ± 9.66	62.71 ± 13.68
Be (mg/kg)	1.94 ± 0.28	2.07 ± 0.34	1.91 ± 0.39
Ba (mg/kg)	521.43 ± 130.31	439.76 ± 50.23	436.76 ± 27.28

Note: PJ stands for Panjin, DG stands for Donggang, and YK stands for Yingkou. Asterisks (*) and letters indicate significant differences among groups.

## Data Availability

The original contributions presented in this study are included in the article/Supplementary Material. Further inquiries can be directed to the corresponding author.

## Supplementary Materials

The following supporting information can be downloaded at: https://www.mdpi.com/article/10.3390/foods15142499/s1, Figure S1: Identification and annotation of metabolites in all test brown rice samples; Figure S2: Detailed heatmap of differential metabolites in rice grains clustered based on biomarkers acquired under positive ion mode; Figure S3: Detailed heatmap of differential metabolites in rice grains clustered based on biomarkers acquired under negative ion mode; Table S1: Sampling regions of the test rice. Table S2: The chromatographic gradient elution program.
