# Supplementary material for: Non-Targeted Metabolomics Reveals the Metabolic Differentiation of Rice from Adjacent Small-Scale Producing Areas and Its Response to Climatic and Soil Factors
Source: Foods. 2026 Jul 15;15(14):2499. doi: 10.3390/foods15142499 (PMC13408526; doi:10.3390/foods15142499)
Supplement: Supplementary file 1 [file foods-15-02499-s001.zip › foods-4406787-supplementary.pdf]

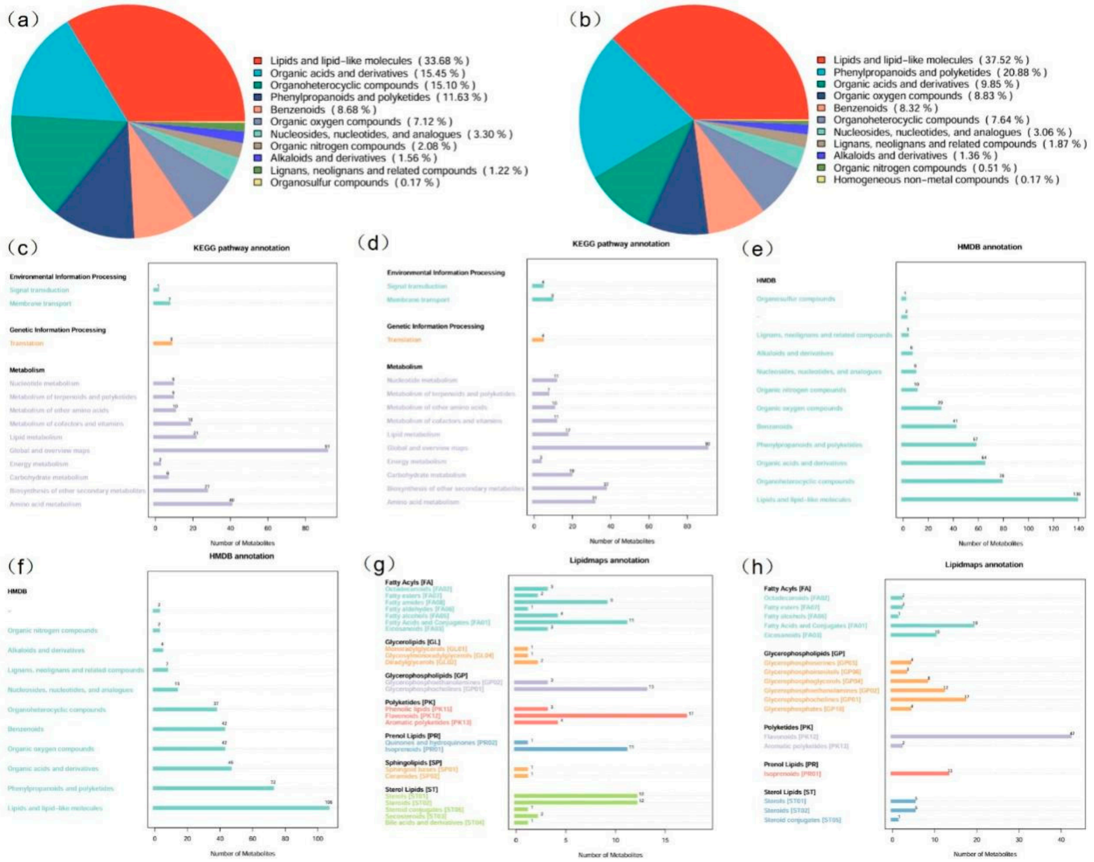

Figure S1 Identification and annotation of metabolites in all test brown rice samples

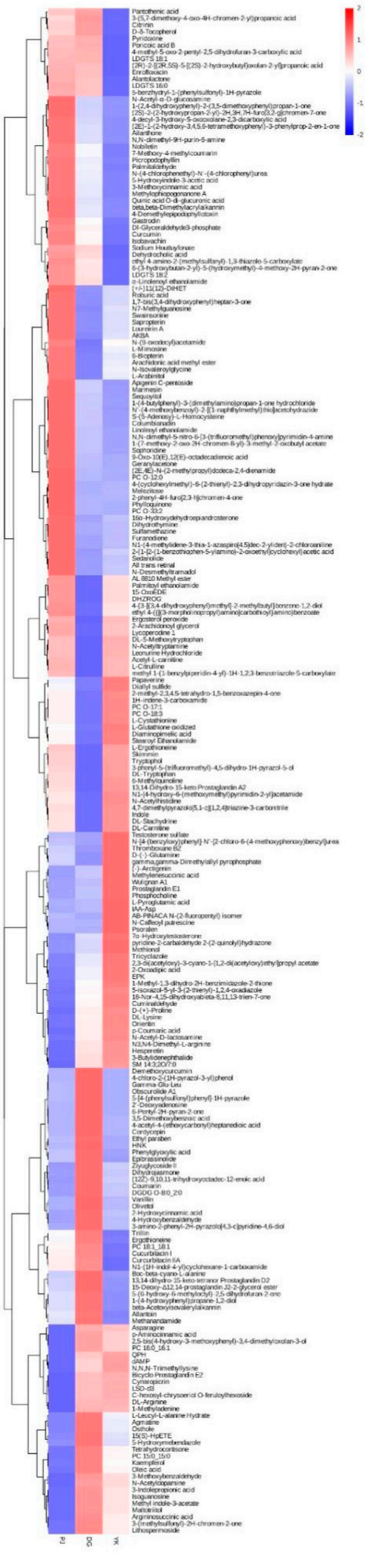

Figure S2 Detailed heatmap of differential metabolites in rice grains clustered based on biomarkers acquired under positive ion mode

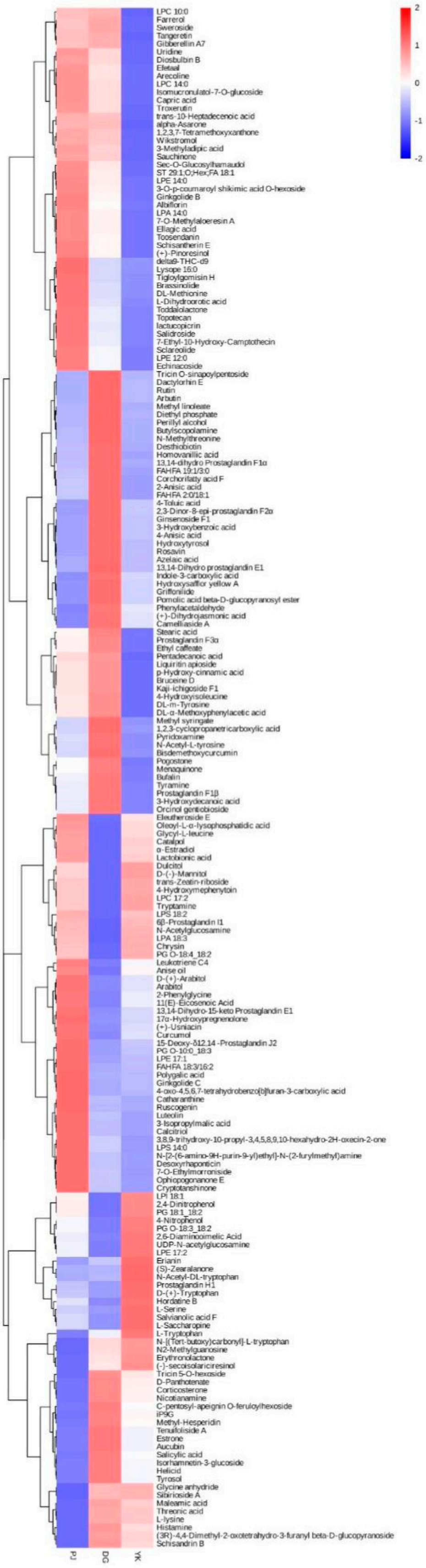

Figure S3 Detailed heatmap of differential metabolites in rice grains clustered based on biomarkers acquired under negative ion mode

Table S1. Sampling regions of the test rice.

Sample Number	Sampling site	Sampling year	Sample variety
PJ1	Liujia Village, Tangjia Town, Dawa District, Panjin City	2023	Yanfeng 47
PJ2	Liujia Village, Tangjia Town, Dawa District, Panjin City	2024	Yanfeng 47
PJ3	Liujia Village, Tangjia Town, Dawa District, Panjin City	2025	Yanfeng 47
PJ4	Sanjiazi Village, Chengjiao Township, Dawa Subdistrict, Panjin City	2023	Yanfeng 47
PJ5	Sanjiazi Village, Chengjiao Township, Dawa Subdistrict, Panjin City	2024	Yanfeng 47
PJ6	Sanjiazi Village, Chengjiao Township, Dawa Subdistrict, Panjin City	2025	Yanfeng 47
PJ7	Xinsheng Subdistrict, Xinglongtai District, Panjin City	2023	Yanfeng 47
PJ8	Xinsheng Subdistrict, Xinglongtai District, Panjin City	2024	Yanfeng 47
PJ9	Xinsheng Subdistrict, Xinglongtai District, Panjin City	2025	Yanfeng 47
DG1	Yangjiatun Village, Changshan Town, Donggang City	2023	Yanfeng 47
DG2	Yangjiatun Village, Changshan Town, Donggang City	2024	Yanfeng 47
DG3	Yangjiatun Village, Changshan Town, Donggang City	2025	Yanfeng 47
DG4	Linjiapu Village, Qianyang Town, Donggang City	2023	Yanfeng 47
DG5	Linjiapu Village, Qianyang Town, Donggang City	2024	Yanfeng 47
DG6	Linjiapu Village, Qianyang Town, Donggang City	2025	Yanfeng 47
DG7	Village, Qianyang Town, Donggang City	2023	Yanfeng 47
DG8	Village, Qianyang Town, Donggang City	2024	Yanfeng 47
DG9	Village, Qianyang Town, Donggang City	2025	Yanfeng 47
YK1	Changtun Village, Qikou Town, Dashiqiao City, Yingkou City	2023	Yanfeng 47
YK2	Changtun Village, Qikou Town, Dashiqiao City, Yingkou City	2024	Yanfeng 47
YK3	Changtun Village, Qikou Town, Dashiqiao City, Yingkou City	2025	Yanfeng 47
YK4	Liansantun Village, Gaokan Town, Dashiqiao City, Yingkou City	2023	Yanfeng 47
YK5	Liansantun Village, Gaokan Town, Dashiqiao City, Yingkou City	2024	Yanfeng 47
YK6	Liansantun Village, Gaokan Town, Dashiqiao City, Yingkou City	2025	Yanfeng 47
YK7	Shifo Village, Shifo Town, Dashiqiao City, Yingkou City	2023	Yanfeng 47
YK8	Shifo Village, Shifo Town, Dashiqiao City, Yingkou City	2024	Yanfeng 47
YK9	Shifo Village, Shifo Town, Dashiqiao City, Yingkou City	2025	Yanfeng 47

Note: PJ stands for Panjin, DG stands for Donggang, and YK stands for Yingkou.

Table S2. The chromatographic gradient elution program

Time (min)	A%	B%
0	98	2
1.5	98	2
3	15	85
10	0	100
10.1	98	2
11	98	2
12	98	2
